# Complete genome sequence of *Rothia mucilaginosa* D1, a genetically amenable strain

**DOI:** 10.1128/mra.00564-25

**Published:** 2025-09-23

**Authors:** Hemendra P.S. Dhaked, Isabella Andonie, Bo Yang, Vinayka M. Joshi, Zezhang T. Wen

**Affiliations:** 1 Department of Oral and Craniofacial Biology, School of Dentistry, Louisiana State University Health Sciences Center 12258 , New Orleans, Louisiana, USA; 2 Department of Periodontics, School of Dentistry, Louisiana State University Health Sciences Center 12258 , New Orleans, Louisiana, USA; 3 Department of Microbiology, Immunology and Parasitology, School of Medicine, Louisiana State University Health Sciences Center 12258 , New Orleans, Louisiana, USA; University of Maryland, School of Medicine, Baltimore, Maryland, USA

**Keywords:** *Rothia mucilaginosa*, genome, biofilm formation

## Abstract

Here, we report the complete genome sequence of *Rothia mucilaginosa* D1. Unlike many other strains that have been sequenced, *R. mucilaginosa* D1, a clinical isolate, is genetically amenable. The complete sequencing of the genome provides potential for further studies on the genetics and pathophysiology of this emerging pathobiont.

## ANNOUNCEMENT

*Rothia mucilaginosa*, one of the most abundant bacterial species in the oral cavity and the upper-respiratory tract, is associated with various acute and chronic diseases, including dental caries, endodontitis, candidiasis, and pneumonia (1), although its exact role in health and disease remains unclear. The lack of genetic transformability remains the major obstacle in the study of *Rothia* pathophysiology. Unlike many other strains that have been sequenced (www.homd.org) (2, 3), *R. mucilaginosa* D1 is genetically amenable.

*R. mucilaginosa* D1 was isolated from a patient’s saliva sample (LSUHSC-NO IRB# 7257) on selective medium as described by Tsuzukibashi et al. (4, 5). For genome sequencing, *R. mucilaginosa* D1 was grown in Brain Heart Infusion broth aerobically at 37°C and harvested when its optical density reached 0.4 at 600 nm. Its genomic DNA was extracted using a ZymoBIOMICS DNA Miniprep Kit. Sequencing and *de novo* assembly were carried out at the SeqCenter (Pittsburgh, PA) under default parameters for all software unless otherwise specified. Briefly, the genomic DNA was fragmented to 40–50 kb using a Megaruptor 3 instrument; sequencing library was prepared using the PacBio SMRTbell Prep Kit 3.0 (PacBio.com, PN: 102-182-700); post-library binding, cleanup, and polymerase treatment were performed with the Revio reagent kit (PacBio.com, PN: 102-739-100); and sequencing was performed using the PacBio Revio platform. Raw sequencing data underwent initial processing with Lima (https://github.com/PacificBiosciences/pbbioconda) for deduplication, adapter trimming, and quality control; BAM files were converted to FastQ format using SamTools (6). A total of 1,082,143 PacBio HiFi reads with an average length of 9,336.5 were analyzed via Flye (v2.9.2) with parameters set as -asm-coverage 50 -genome size 6 Mbp -pacbio hifi read-error 0.02 (7). Eligible contigs were circularized using Circulator (v1.5.5) using all pipeline and internal *minimus2*-based steps, followed by start position adjustment to *dnaA* to achieve a complete chromosome (8). The final assembly was functionally annotated using the NCBI Prokaryotic Genome Annotation Pipeline (v6.10) under default settings (9); assembly quality metrics, including contiguity and completeness, were assessed using QUAST (v5.2.0) and reported in a tsv format (10).

The genome of *R. mucilaginosa* D1 is 2,246,281 bp long with a GC content of 59.5%. It is predicted to encode a total of 1,787 coding sequences. It contains *comEA* with a potential role in DNA transport and probably competence development (11). It has multiple genes for phosphoenolpyruvate:sugar phosphotransferase systems, including lactose, glucose, beta-glucoside, mannose, and fructose, along with three non-specific sugar transporters. It contains a NAD-dependent glutamate dehydrogenase and two copies of type I glutamate-ammonia ligases, aka glutamine synthetases. *R. mucilaginosa* D1 is catalase-negative. The complete sequence and annotation presented here should augment future study of this organism in addition to providing resources for genetic manipulation.

## Data Availability

The genome sequence of *R. mucilaginosa* D1 has been deposited in GenBank under the accession number CP188231.1. The raw sequencing reads are available in the Sequence Read Archive under the accession number SRR33389990. These records are associated with BioProject PRJNA1254216 and BioSample SAMN48100297.

## References

[1] Fatahi-Bafghi M. 2021. Characterization of the Rothia spp. and their role in human clinical infections. Infect Genet Evol 93:104877. doi:10.1016/j.meegid.2021.104877doi:33905886

[2] Nambu T, Tsuzukibashi O, Uchibori S, Mashimo C. 2015. Complete genome sequence of Rothia mucilaginosa strain NUM-Rm6536, isolated from a human oral cavity. Genome Announc 3:e01122-15. doi:10.1128/genomeA.01122-15 PMC4591313 26430041

[3] Oliveira IMF, Ng DYK, van Baarlen P, Stegger M, Andersen PS, Wells JM. 2022. Comparative genomics of Rothia species reveals diversity in novel biosynthetic gene clusters and ecological adaptation to different eukaryotic hosts and host niches. Microb Genom 8:mgen000854. doi:10.1099/mgen.0.000854 36165601 PMC9676035

[4] Kobayashi T, Uchibori S, Tsuzukibashi O, Goto H, Aida M. 2012. A selective medium for Rothia mucilaginosa and its distribution in oral cavities. J Microbiol Methods 91:364–365. doi:10.1016/j.mimet.2012.09.011 22995714

[5] Tsuzukibashi O, Uchibori S, Kobayashi T, Umezawa K, Mashimo C, Nambu T, Saito M, Hashizume-Takizawa T, Ochiai T. 2017. Isolation and identification methods of Rothia species in oral cavities. J Microbiol Methods 134:21–26. doi:10.1016/j.mimet.2017.01.005 28082174

[6] Li H, Handsaker B, Wysoker A, Fennell T, Ruan J, Homer N, Marth G, Abecasis G, Durbin R. 2009. The sequence alignment/map format and SAMtools. Bioinformatics 25:2078–2079. doi:10.1093/bioinformatics/btp352 19505943 PMC2723002

[7] Freire B, Ladra S, Parama JR. 2022. Memory-efficient assembly using flye. IEEE/ACM Trans Comput Biol Bioinform 19:3564–3577. doi:10.1109/TCBB.2021.3108843 34469305

[8] Hunt M, Silva ND, Otto TD, Parkhill J, Keane JA, Harris SR. 2015. Circlator: automated circularization of genome assemblies using long sequencing reads. Genome Biol 16:294. doi:10.1186/s13059-015-0849-0 26714481 PMC4699355

[9] Tatusova T, DiCuccio M, Badretdin A, Chetvernin V, Nawrocki EP, Zaslavsky L, Lomsadze A, Pruitt KD, Borodovsky M, Ostell J. 2016. NCBI prokaryotic genome annotation pipeline. Nucleic Acids Res 44:6614–6624. doi:10.1093/nar/gkw569 27342282 PMC5001611

[10] Gurevich A, Saveliev V, Vyahhi N, Tesler G. 2013. QUAST: quality assessment tool for genome assemblies. Bioinformatics 29:1072–1075. doi:10.1093/bioinformatics/btt086 23422339 PMC3624806

[11] Seitz P, Pezeshgi Modarres H, Borgeaud S, Bulushev RD, Steinbock LJ, Radenovic A, Dal Peraro M, Blokesch M. 2014. ComEA is essential for the transfer of external DNA into the periplasm in naturally transformable Vibrio cholerae cells. PLoS Genet 10:e1004066. doi:10.1371/journal.pgen.1004066 24391524 PMC3879209

